# Progress with the Learning Health System 2.0: a rapid review of Learning Health Systems’ responses to pandemics and climate change

**DOI:** 10.1186/s12916-024-03345-8

**Published:** 2024-03-22

**Authors:** Carolynn L. Smith, Georgia Fisher, Putu Novi Arfirsta Dharmayani, Shalini Wijekulasuriya, Louise A. Ellis, Samantha Spanos, Genevieve Dammery, Yvonne Zurynski, Jeffrey Braithwaite

**Affiliations:** 1 Centre for Healthcare Resilience and Implementation Science, Australian Institute of Health Innovation, Macquarie University, 75 Talavera Road, North Ryde 2113, Sydney, Australia; 2 NHMRC Partnership Centre for Health System Sustainability, Macquarie University, 75 Talavera Road, North Ryde 2113, Sydney, Australia

**Keywords:** Learning Health Systems, Climate change, Pandemics, COVID-19

## Abstract

**Background:**

Pandemics and climate change each challenge health systems through increasing numbers and new types of patients. To adapt to these challenges, leading health systems have embraced a Learning Health System (LHS) approach, aiming to increase the efficiency with which data is translated into actionable knowledge. This rapid review sought to determine how these health systems have used LHS frameworks to both address the challenges posed by the COVID-19 pandemic and climate change, and to prepare for future disturbances, and thus transition towards the LHS2.0.

**Methods:**

Three databases (Embase, Scopus, and PubMed) were searched for peer-reviewed literature published in English in the five years to March 2023. Publications were included if they described a real-world LHS’s response to one or more of the following: the COVID-19 pandemic, future pandemics, current climate events, future climate change events. Data were extracted and thematically analyzed using the five dimensions of the Institute of Medicine/Zurynski-Braithwaite’s LHS framework: *Science and Informatics*, *Patient-Clinician Partnerships, Continuous Learning Culture*, *Incentives*, and *Structure and Governance*.

**Results:**

The search yielded 182 unique publications, four of which reported on LHSs and climate change. Backward citation tracking yielded 13 additional pandemic-related publications. None of the climate change-related papers met the inclusion criteria. Thirty-two publications were included after full-text review. Most were case studies (*n* = 12, 38%), narrative descriptions (*n* = 9, 28%) or empirical studies (*n* = 9, 28%). *Science and Informatics* (*n* = 31, 97%), *Continuous Learning Culture* (*n* = 26, 81%), *Structure and Governance* (*n* = 23, 72%) were the most frequently discussed LHS dimensions. *Incentives* (*n* = 21, 66%) and *Patient-Clinician Partnerships* (*n* = 18, 56%) received less attention. Twenty-nine papers (91%) discussed benefits or opportunities created by pandemics to furthering the development of an LHS, compared to 22 papers (69%) that discussed challenges.

**Conclusions:**

An LHS 2.0 approach appears well-suited to responding to the rapidly changing and uncertain conditions of a pandemic, and, by extension, to preparing health systems for the effects of climate change. LHSs that embrace a continuous learning culture can inform patient care, public policy, and public messaging, and those that wisely use IT systems for decision-making can more readily enact surveillance systems for future pandemics and climate change-related events.

**Trial registration:**

PROSPERO pre-registration: CRD42023408896.

**Supplementary Information:**

The online version contains supplementary material available at 10.1186/s12916-024-03345-8.

## Background

The COVID-19 pandemic presented many multi-faceted challenges to health systems worldwide, stimulating rapid responses to cope with increasing numbers and new types of patients [1–3]. Climate change is already having similar effects [4, 5], causing patient numbers to surge immediately following climate-related disasters, such as hurricanes and heatwaves. Over longer time periods, global warming will create more pressure on health systems through new emerging infectious and vector-borne diseases, the effects of pollution, and the exacerbation of chronic conditions [6–9]. To strengthen their resilience to future pandemics and climate-related disasters, health systems will need to rapidly integrate new evidence into healthcare practices and health policies [5, 10, 11]. A promising method of doing so is to transform health systems into Learning Health Systems (LHS) [12, 13], where “science, informatics, incentives, and culture are aligned for continuous improvement and innovation, with best practices seamlessly embedded in the care process, patients and families active participants in all elements, and new knowledge captured as an integral by-product of the care experience” [14].

The LHS framework proposed in 2007 by the Institute of Medicine (IoM) [12] (now known as the National Academy of Medicine) comprised four dimensions: *Science and Informatics*, *Patient-Clinician Partnerships*, *Incentives*, and a *Continuous Learning Culture*. Under this framework, *Science and Informatics* encompasses information technology (IT) systems needed to capture, collate, and disseminate data (e.g., electronic health records (eHRs), data warehouses and repositories, dashboards and decision-support tools) to produce actionable knowledge. *Patient–Clinician Partnerships* envisions patients, families, carers, and the broader public as partners in the co-design and development of programs. *Incentives* are aligned for continuous improvement and promote transparency around outcomes, costs, safety and quality to inform patient and clinician decisions and choices. A *Continuous Learning Culture* is facilitated by leadership and supported by staff capability and skills to create a feedback loop wherein the system is continuously refined by new knowledge generated from patients and research (such as genomics, proteomics, and clinical trials research) [13, 15, 16]. In 2020, the framework was refined by our team to include *Structure and Governance*, which encompasses the policies, regulations, and governance of the health system [17].

Health systems that have begun to approximate the elements in this LHS framework as a routine way of working should have greater capacity to adapt and respond to the challenges posed by pandemic- and climate change-related impacts. Prior to, and over the course of, the COVID-19 pandemic, an increasing number of health systems have embraced LHS ideas and principles to more rapidly turn data into knowledge that can inform best practice [17–20]. The NHS England’s Nightingale Hospital London (UK) and New South Wales Health’s Critical Intelligence Unit (Australia) are two prime examples of health systems rapidly applying LHS principles in response to the COVID-19 pandemic [1, 21].

To the best of our knowledge, there are no reviews which examine how these real-world LHSs (i.e., those health systems that have strategically advanced to operate via LHS framework principles) are responding on the ground to both climate change-related events and pandemics, current or future. In this rapid review, we aimed to:

1. Identify and describe how the LHS framework is being used to address the challenges to the health system that are currently posed by the COVID-19 pandemic and climate change.
2. Understand how LHS frameworks are being used to prepare for future challenges to the health system that will be created by pandemics and climate change.

Since the original proposal by the IoM, the published literature in this area has grown rapidly but much of the literature is case-based or remains theoretically focused on advancing the LHS as a concept [22–26]. While the latter does provide important information on LHS progress, it is equally important that our understanding be informed by empirical investigations of LHSs.

In early 2023, Braithwaite and colleagues coined the label LHS 2.0, referring to a healthcare provider whose model of care was increasingly accomplished at marshaling information, data, and intelligence, and at using them to prepare for future pandemics, and the pressures, crises, and sequelae associated with climate change [27]. This rapid review is a test of how far that model has been or is being realized in real world settings.

## Methods

We performed a rapid review of empirical studies on climate change, pandemics and human health systems using the methods described in the Cochrane Guidelines for Rapid Reviews [28, 29] and guided by the Preferred Reporting Items for Systematic reviews and Meta-Analyses (PRISMA [28]). The review protocol was pre-registered on PROSPERO: CRD42023408896.

### Search strategy

The PubMed, Embase, and Scopus databases were searched on the 14th of March 2023 for articles published in English from the 1st of January 2018 to 14th March 2023. Two searches were run in each database: one for pandemics and one for climate change. The two searches combined the term (learning health* system*) with terms associated with either pandemics or climate change respectively. The search strategy for the list of climate-related events was drawn from a systematic review published in *The Lancet Planetary Health* [30]*.* The full search strategies are detailed in Additional file 1: Table S1.

### Inclusion criteria

#### Pandemics

Studies were included if they discussed a real-world LHS in relation to the current COVID-19 pandemic, past or future pandemics, in any health system setting, in any country (e.g., low, middle, or high income).

#### Climate change

Studies were included if they discussed the LHS’s response to current or future climate change threats or climate-related events (e.g., tropical cyclones, floods, heat waves, vector borne diseases, droughts and dust storms), in any health system setting, in any country [30].

### Exclusion criteria

Studies were excluded if they were not peer reviewed; not written in English; were a commentary, perspective, or other opinion piece; did not identify the health system as an LHS; or did not explicitly discuss an LHS’s response to pandemics (past, current, or future) or climate change or discussed non-climate change related events (e.g., earthquakes, blackouts, erosion (secondary to changes in rainfall), tsunamis) [30].

### Screening

#### Title and abstract

Four reviewers (GF, SW, PNAD, CLS) screened 20% of titles and abstracts against the study inclusion criteria and then assessed their agreement and conducted a conflict resolution. The same four reviewers then each screened a quarter of the remaining abstracts. After this, one reviewer screened all excluded abstracts to confirm the decision to exclude and then resolved conflicts with the review team.

#### Full text

Four reviewers (GF, SW, PNAD, CLS) screened 20% of full texts and then assessed their agreement and conducted a conflict resolution. Each reviewer then screened a quarter of the remaining full texts. One reviewer screened all excluded full-text articles to confirm the decision to exclude and then resolved questions with the review team. Additional relevant publications were identified from the included publications (backward citation searching [31]) and then underwent the same screening process.

### Data extraction

Four authors (CLS, GF, PNAD, SW) conducted the data extraction process. A custom data extraction form in REDCap (version 10.0.6 [32]) was piloted on three included articles, refined, and approved by the same four authors (Additional file 1: Table S2). The remaining articles were then divided among the four authors to complete the data extraction. Extracted data were checked by another reviewer for correctness and completeness. The four authors resolved any outstanding queries through discussion. Extracted data included information about each LHS, including health system sector (e.g., international, state), setting (e.g., hospital network, primary care), rurality, host country, and OECD classification, as well as any definition of an LHS used (along with any sources cited). Details of the actions taken in respond to or to prepare for pandemics or climate change in each included study were extracted. Opportunities and challenges created by the events to advancing or hindering the development of the LHS were also extracted.

### Quality assessment of included articles

To assess the scope and quality of included publications, three quality appraisal tools were used depending on methodology used in the publication: the Mixed-Methods Appraisal Tool (MMAT [33]), the Scale for the Assessment of Narrative Review Articles (SANRA) [34]), and the Joanna Briggs Institute (JBI) critical appraisal checklist for systematic reviews and research synthesis [35]. The authors that extracted data assessed the quality of included studies. Papers scored using MMAT were given a mark out of five (0–1 = high quality, 2–3 = moderate quality, 4–5 = low quality). Papers assessed using SANRA were given a mark out of 6 (0–1 = high quality, 2–3 = moderate quality, 4–6 = low quality). Papers scored using the JBI tool were marked out of 11 (0–3 = high quality, 3–6 = moderate quality, > 6 = low quality).

### Data synthesis

#### LHS setting, definitions, and frameworks

Data that described each LHS setting were summarized descriptively via counts and percentages of included studies. We also calculated counts of unique references used to cite each article’s definition of an LHS and recorded the most quoted definitions to provide an understanding of how LHSs were conceptualized in real-world health systems. Additionally, we calculated counts of the number of studies that reported using an LHS framework to guide the LHS’s pandemic or climate change response, and whether these frameworks were developed in response to climate change or pandemics, adapted from an existing framework, or were an existing framework that had not been modified from the original.

#### LHS responses to pandemics and climate change

Data that described LHS responses to pandemics and climate change were synthesized using a deductive framework approach. The coding scheme was aligned with the LHS framework for an LHS proposed by the IoM (2013 [13]) and expanded by Zurynski et al. (2020 [17]), comprising five dimensions: *Science and Informatics*, *Patient-Clinician Partnerships, Continuous Learning Culture*, *Incentives*, and *Structure and Governance*. A single author coded data in each LHS dimension. Then, the same author inductively generated key sub-themes of the actions taken by included studies under each LHS dimension. These sub-themes and their associated data were reviewed by four authors (CLS, GF, PNAD, SW) and any disagreements were resolved.

#### Opportunities and challenges posed by pandemics and climate change to the development of the LHS

Data that described the opportunities and challenges posed by pandemics and climate change to the development or advancement of the LHS were synthesized using an inductive thematic analysis approach. Themes were generated by four members of the review team (CLS, GF, PNAD, SW) and then consolidated based on qualitative codes through group discussion and cross checking among the four team members. Data were then coded to each theme.

## Results

**Fig. 1:**
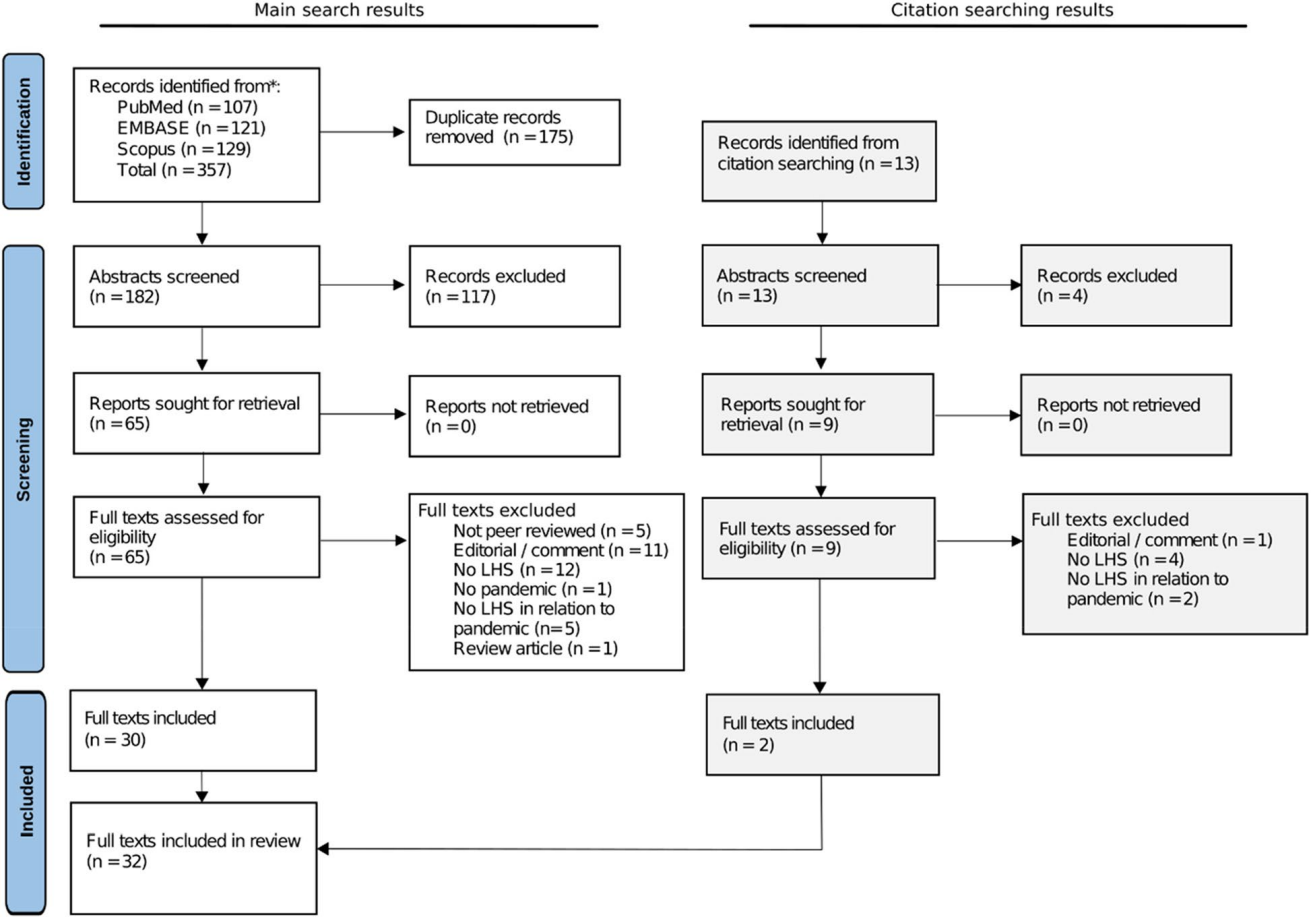
PRISMA flow diagram for study selection process. LHS Learning health system

The search on pandemics and LHSs yielded 353 results, while the search on climate change and LHSs yielded four results. After duplicate removal, the title and abstract of 182 articles were screened against the inclusion and exclusion criteria. Of these, 117 articles did not meet eligibility criteria, including the four papers identified from the climate change and LHS search. Sixty-five full texts were assessed for eligibility. Thirteen additional papers were identified via backwards citation searching of included full texts and underwent the same screening process. Thirty-two papers were included after the final screening (Fig. 1).

Of the 32 included publications that described an LHS response to a pandemic, 12 (38%) were case studies, 9 were narrative descriptive articles (28%), 9 were empirical studies (28%), 1 was a protocol (3%), and 1 was a policy-focused publication (3%). Two papers discussed “long COVID” [31, 36] and one paper discussed the COVID-19 pandemic and another pandemic disease (tuberculosis [37]). The other 29 papers (90%) discussed experiences during the COVID-19 pandemic.

The 32 included studies covered nine countries: the USA (*n* = 18, 56%), Canada (*n* = 5, 16%), UK (*n* = 3, 9%), and one paper from each of France, Guinea, Kenya, Nigeria, South Africa, and Spain (3% for each paper). Settings included hospitals and medical centers (*n* = 7, 22%), primary care (*n* = 5, 16%), large health networks (*n* = 11, 34%), such as the US’s Veterans Health Administration, and several community health services (e.g., prevention, vaccination) (*n* = 7, 22%). Some studies covered multiple settings. Details of all included LHSs are reported in Table 1 and Additional file 1: Table S3.

**Table 1:** Included study summary

First author (year) Country	LHS health sector	LHS Health Setting			
		**Organization setting**	**Location**	**OECD**	**Country**
Case study					
Allen (2021) [38] USA	Local	Research institute	N/A	High	USA
Bakshi (2021) [39] USA	International	Hospital network	NR	High	USA, International
Braganza (2022) [40] USA	National	Hospital network	NR	High	USA
Brunet (2022) [41] Canada	State	Academic health centers	NR	High	Canada
English (2021) [42] Kenya	National	Hospital network	NR	Low-mid	Kenya
Fox (2021) [43] UK	Digital	Hospital, research institute	Urban	High	UK
Groot (2022) [2] Canada	State	NR	Urban Regional	High	Canada
Levin (2022) [44] Canada	State	Health system, policymakers, advisory groups	NR	High	Canada
McCreary (2022) [45] USA	State	Hospital, Community service, Primary care,	Urban Regional Rural	High	USA
Millimouno (2023) [46] Guinea	National	Whole health system	Urban Regional Rural	Low	Guinea
Vahidy (2021) [47] USA	Community	Hospital network, primary care	Urban	High	USA
Vinson (2021) [48] USA	National	Hospital network, community service	NR	High	USA
Narrative descriptive study					
Anderson (2022) [49] USA	Community	Hospital, outpatient, university medical system	Urban	High	USA
Atkins (2022) [50] USA	National	Hospital network	Urban Regional	High	USA
Daniel (2022) [51] France	National	Hospital network	Urban	High	France
Foraker (2021) [52] USA	Whole health sector	Hospital network, research institute, university	Urban	High	USA
Gustavson (2022) [31] USA	National	Hospital network, community service, primary care, rehabilitation, specialty care	Urban Regional Rural	High	USA
Hunt (2021) [53] USA	International	Learning network	N/A	All	International
Ros (2021) [54] Spain	International	Whole health system	NR	High	Spain, US, Italy
Saleh (2021) [55] Nigeria	National	Community service, primary care, local government facilities	Urban Regional Rural	Low-mid	Nigeria
Wood (2021) [56] USA	National	Non-profit organization	N/A	High	USA
Empirical study					
Cassidy (2022) [57] Canada	Community	Hospital	Urban Rural	High	Canada
Dash (2022) [58] USA	Community	Hospital	Urban	High	USA
Groot (2022) [59] Canada	State	Health system, policy Makers, advisory groups	NR	High	Canada
McCreary (2022) [60] USA	State	Hospital network	NR	High	USA
McCreary (2022) [61] USA	State	Hospital, community service, primary care	Urban Regional Rural	High	USA
Polancich (2021) [62] USA	Community	Hospital, academic health center	Urban	High	USA
Tai-Seele (2022) [63] USA	Community	Hospital, community service	Urban	High	USA
UPMC REMAP-COVID Group (2021) [64] USA	National	Hospital network	Urban	High	USA
van Rensburg (2022) [37] South Africa	Community	Community service	Regional	Mid-upper	South Africa
Policy focused					
Sheikh (2021) [65] UK	National	Whole health system	Urban Regional Rural Remote	High	UK
Study protocol					
Sivan (2022) [36] UK	Community	Community service, primary care, specialist clinics	NR	High	UK

*LHS* Learning Health System, *OECD* Organisation for Economic Cooperation and Development Status, *USA* United States of America, *UK* United Kingdom, *N/A* not applicable, *NR* not reported

### Quality assessment of included articles

The SANRA was used to appraise 15 papers and the MMAT was appropriate for 17 papers. The JBI tool was not appropriate for any papers. Most of the papers were appraised as high (*n* = 15, 47%) or moderate quality (*n* = 16, 50%), only one paper was of low quality (3%) [39]. This paper was still included because it provided specific data about real-world LHS responses to the COVID-19 pandemic (Additional file 1: Table S4).

### LHS definitions and frameworks

Twenty papers included a definition of an LHS [2, 31, 37, 38, 40, 41, 44, 46, 48, 52, 54, 57–65] (Table 2). Twenty-seven unique references were used to cite these LHS definitions. The most cited references were those originating from the IoM (cited in *N* = 9 papers, 28%) [31, 40, 44, 57–60, 62, 65] or with authorship from Friedman (cited in *N* = 7 papers, 22%) [41, 43, 52, 54, 58, 63, 65].

Of 17 studies (53%) that reported use of an LHS framework, 11 used existing frameworks that were not explicitly modified from their original structure [37, 38, 40, 41, 43, 46, 48, 51, 56, 57, 62], 4 adapted an existing framework in response to the COVID-19 pandemic [44, 49, 59, 64], and 2 developed new frameworks [52, 54] (Table 2).

**Table 2:**
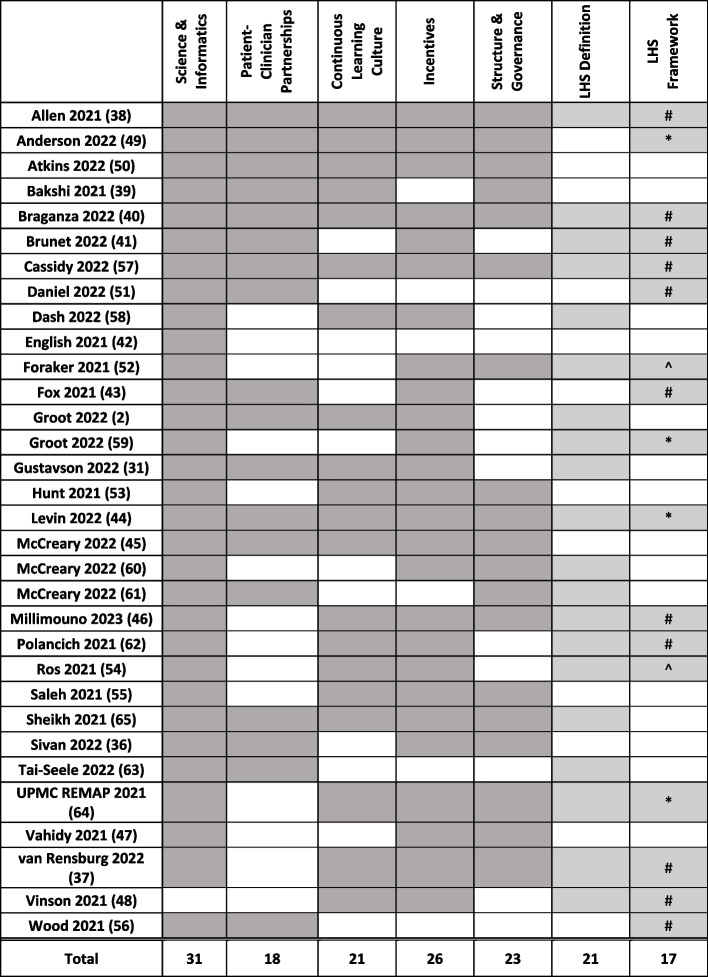
Included publications coverage of LHS dimensions, and LHS definitions and frameworks [38, 49, 50, 39–41, 57, 51, 58, 42, 52, 43, 2, 59, 31, 53, 44, 45, 60, 61, 46, 62, 54, 55, 65, 36, 63, 64, 47, 37, 48, 56]

Gray-shaded boxes indicate that the publication included a dimension, definition, or framework. In the Framework column, number sign indicates an existing framework, asterisk indicates an adapted framework, and circumflex accent indicates a new framework

### Real-world LHSs responses to pandemics categorized by dimension

*Science and Informatics* (*n* = 31, 97%), *Continuous Learning Culture* (*n* = 26, 81%), and *Structure and Governance* (*n* = 23, 72%) were the most frequently discussed LHS dimensions. Incentives (*n* = 21, 66%) and *Patient-Clinician Partnerships* (*n* = 18, 56%) received less attention (Table 2). Twenty-nine papers (91%) discussed benefits or opportunities arising from the societal and health system conditions created by the COVID-19 pandemic compared to 22 papers (69%) which discussed challenges presented by a pandemic to the development or advancement of an LHS.

#### Science and Informatics

Thirty-one articles (97%) described an LHSs response to current or future pandemics that involved the *Science and Informatics* dimension of the framework (Table 2). Within this dimension, four sub-themes were discerned (Table 3). The most frequently discussed sub-theme was the use of *healthcare information systems* (*n* = 26, 84%), including eHRs, machine learning/AI, and clinical prediction/decision making tools [31, 36–38, 40–44, 46, 47, 49–52, 54–58, 60, 61, 63–65]. *Data-driven research and knowledge translation* was reported in 21 articles (68%) [2, 37–42, 44–47, 49–52, 55–57, 59, 61, 65]. For example, data were integrated into dashboards and modeling tools to facilitate clinical decision-making for COVID-19 patients within the University of Montreal’s LHS [41]. Sixteen articles (52%) explicitly mentioned a range of *digital healthcare services* in the response to pandemic, which included remote consultation programs, mobile health applications, websites, or smart devices to deliver patient care [2, 36, 39, 41, 43, 47, 49, 51, 53, 55, 57, 58, 62–65]. *Health data management* (*n* = 15, 48%), which was the least discussed sub-theme, covered the use of data warehouses, repositories, databases, registries, and data linkage to manage, organize, and integrate health data [2, 44, 45, 47, 50–52, 54, 56–58, 60, 61, 64, 65]. While these sub-themes were common across included articles, their individual application varied according to context. For example, both Ros et al. [54] and Vahidy et al. [47] described a LHS that was predicated on the creation and use of standardized databases. Notably, Ros et al. had access to key stakeholders across multiple countries and thus formed a transatlantic cooperation that aimed to change national policies and prospectively create a standard data model. Conversely, Vahidy et al. described an LHS in a single organization, and thus were able to rapidly create an extensive data repository populated via their electronic medical records, where data was standardized both prospectively and retrospectively.

**Table 3:** Sub-themes inductively developed in thematic analysis of LHS responses to pandemics

Theme	*n*	Example text from article	Refs
Subtheme			
Definition			
*Science and Informatics*			
Heath information systems * Centralizing electronic data processing, storage, linking, and prediction (eHR/eMR, clinical prediction/decision, routine data collection, machine learning/AI)*	26	“The technology team programmed automated eHR alerts which prompt clinicians to complete the form when a SARS-CoV-2 test is ordered, a COVID positive isolation flag is entered, or a COVID order set is initiated, to capture basic data for surveillance reporting to public health authorities. Clinicians can suppress the form if they do not consider their patient to have active COVID-19.” [64]	[31, 36–38, 40–47, 49–52, 54–58, 60, 61, 63–65]
Data-driven research and knowledge translation * Using data to automate or drive decision making and practice change (dashboard, policy, report, publication, meeting agenda based on evidence/data)*	21	“…The dashboard was generated from the database and offered a snapshot of number of questions, their status (search in progress, review in progress, completed, update in progress, updated review, cancelled, review delayed).” [2]	[2, 37–42, 44–47, 49–52, 55–57, 59, 61, 65]
Digital healthcare services * Using information and communication technologies to capture and monitor patient data (remote consultation programs, the utilization of mobile apps, software, websites, smart devices)*	16	“Some of the key COVID-19 Yorkshire Rehabilitation Scale questions have been embedded into a bespoke digital platform which monitors general health, The patient completes the questionnaire on a smartphone application and the clinicians access the results on a web portal.” [36]	[2, 36, 39, 41, 43, 47, 49, 51, 53, 55, 57, 58, 62–65]
Health data management * Managing the lifecycle of health data (data warehouse, repositories, database, registry, data linkage)*	15	“… the RRTF decided to develop and actively maintain a registry for COVID-19 surveillance and intrahospital outcomes as a key tangible output of its research-acceleration function.” [47]	[2, 44, 45, 47, 50–52, 54, 56–58, 60, 61, 64, 65]
*Patient–Clinician Partnerships*			
Dissemination * Communicating with the public, Improving patient health literacy*	14	“The trial team also works closely with a team of communications experts to share patient stories, engage with local media to raise awareness, and create patient-facing educational materials.” [45]	[38–41, 43–45, 50, 51, 56, 57, 61, 63, 65]
Engagement * Co-design of programs, Stakeholder consultation*	9	“…the LHS invested in elevating patient voices through focus groups, interviews, and surveys.” [38]	[31, 36, 38, 40, 44, 45, 49, 50, 61]
Inclusiveness * Equitable access to care*	5	“However, the proliferation of telehealth also requires a deliberate focus on health equity.” [39]	[36, 39, 45, 49, 65]
Other * Exclusion of patients* * Implementation theories*	3	"Lay members of the public were not involved when CEST was being developed due to time and financial constraints." [2] “To this end, the growing field of implementation science will be invaluable to the continued maturation and application of clinical prediction rules to patient care.” [50]	[2, 50, 57]
*Incentives*			
Financing * Program financial support for the development/maintenance of the LHS* * Staff financial support for staff training, capacity building,* * Rewards and incentives, such as* * funding for participation*	15	"The KPWA LHS receives programmatic funding from the health system and external funding through research-care delivery partnerships.” [38] “The CoP may also provide learning and growth opportunities, such as clinical or VHA operational fellowships and research grants.” [31] "Best practices in patient engagement, including financial compensation for participation, were incorporated" [44]	[31, 37–40, 44, 46, 49, 53, 55, 57, 58, 62, 65]
Transparency * Information available about decision-making, outcomes, processes, and costs*	8	"To achieve this acceptance, trustworthiness, trust, and transparency regarding rules and ownership for this traceability platform will be mandatory." [54]	[31, 38, 45, 48, 50, 54, 59, 64]
Policies aligned for value * Policies promote measurement and evaluation of programs and improvement of processes*	5	“We measure this by monitoring average time from publication of high quality evidence (e.g., Cochrane reviews, meta-analyses) to publication of an organizational guideline for a practice, and average time from release of an internal evidence-based practice (EBP) guideline to uptake among a percentage of the organization's care teams.” [38]	[38, 46, 48, 54, 55]
Program sustainability * Long term viability of the LHS*	2	“… the CEST initiative experienced financial restrictions which affected its accessibility and sustainability.” [2]	[2, 62]
*Continuous Learning Culture*			
Workforce Experience and training * Facilitating knowledge and skill development in the LHS workforce*	20	"Training programs were rapidly deployed to healthcare professionals to face this new disease, such as for patient transportation, clinical protocol, and personal protective equipment" [41] “To maintain trial awareness, we continuously reach out to hospitals across the system to promote the trial, identify local champions, and address questions" [64] "CoP members and leadership facilitate communication and set up formal collaborations with other non-VHA health care systems to create an intersystem network of collaboration for long COVID care" [31] "…dedicated time to meet to solve problems and support continuous learning" [38]	[2, 31, 37, 38, 40, 41, 43–49, 53, 55, 57, 60, 62, 64, 65]
Empowerment * Championing workforce members to become integral to the LHS*			
Leadership * Formal or informal leadership teams that facilitate a culture of change and learning*			
Capacity * Dedicated time, capacity-building or institutional support in the LHS workforce*			
Continuous refinement and learning * Using feedback and review to inform cycles of quality improvement*	19	“…Once the data are validated, data scientists create report summaries and the evidence is presented to the initiators of the request to inform clinical guideline creation. As a clinical need evolves over time, this process can undergo further iterative cycles to refine guidelines to adapt to a changing environment."[58]	[2, 31, 40, 43–47, 50, 53–55, 57–60, 62, 64, 65]
Communication pathways * Facilitating communication *via* meetings, newsletters, dashboards, or resources*	13	“The distribution of health education resources and the supporting of online training was facilitated by an online webinar for managers and facility trainers, as well as through the establishment of a training support WhatsApp group.” [37]	[2, 31, 36, 37, 44, 47, 48, 53, 55, 57, 58, 60, 64]
Learning collaborations * Learning *via* collaborations between stakeholders or a multidisciplinary team*	12	"Working groups established within each regional health authority inclusive of patients, clinicians, professional services, and researchers." [44]	[2, 31, 36, 37, 44, 47, 52, 55, 57–59, 64]
Structure and Governance			
Leveraging committees * Creating or using established committees to structure pandemic response*	18	To provide rapid responses while preserving research integrity, two system-wide subcommittees were established…..” [47]	[2, 36–38, 40, 44–47, 49, 51, 55, 57, 59–61, 64, 65]
Leveraging supportive collaborations * Formalising collaborations with external organizations to structure the pandemic response*	14	“to enhance coordination and learning as regards public health emergencies, the NCDC collaborated with state governments to set up Public Health Emergency Operations Centres (PHEOCs)." [55]	[2, 40, 44, 49, 52–55, 57, 59–61, 64, 65]
Leveraging state/national policy * Using, modifying, or creating state or national policy in the pandemic response*	12	“Data access for researchers is governed by the policies and procedures dictated by the academic institutions and Ministry of Health.” [44]	[36, 40, 44–47, 50, 54, 55, 61, 64, 65]
Leveraging local policy * Using, modifying, or creating LHS-specific l policies in the pandemic response*	9	“[We] assessed the quality of evidence of new programs and policies submitted as VHA legislative and budget proposals using a novel strength of evidence checklist." [40]	[38–40, 45, 51, 52, 54, 57, 61]
Setting strategic goals and planning * Creation of formal goals or plans for the pandemic response*	6	“We intentionally align[ed] learning activities and opportunities with strategic and operational goals across organizational levels.” [38]	[37, 38, 40, 47, 55, 57]
Funding Financial investment in research and staff * Provision of funding for staff specific to pandemic response*	3	“Research scholars and analytic staff have been hired to support a robust set of outputs to inform care and identify research questions.” [44]	[44, 45, 51]

*n* number of studies. Acronyms within table: *AI* artificial intelligence, *CEST* COVID-19 evidence support team, *COP* community of practice, *eHR* electronic health records, *eMR* electronic medical records, *KPWA* Kaiser Permanente Washington, *NCDC* Nigeria Centre for Disease Control, *RRTF* retrospective research task force, *VHA* US Veterans Health Administration

#### Patient–clinician partnerships

Eighteen papers (56%) covered *Patient-Clinician Partnerships* (Table 2). Four sub-themes were identified within this dimension (Table 3). *Health literacy and public messaging* was the most common subtheme (*n* = 14, 78%) [38–41, 43–45, 50, 51, 56, 57, 61, 63, 65]. For instance, the University of Pittsburgh Medical Center (UPMC) collaborated with communication experts to develop resources that would be accessible to patients and other community members [60]. The importance of *collaboration with patients* (*n* = 9, 50%) [31, 36, 38, 40, 44, 45, 49, 50, 61] and *inclusiveness and equitable access to programs* (*n* = 6, 33%) [36, 39, 45, 49, 61, 65] was also emphasized in this dimension. Two papers (11%) referred to the explicit exclusion of patients and families in decision-making due to either the restrictions enacted in response to the COVID-19 pandemic or the speed at which new evidence was being used within the LHS [2, 57]. One paper (3%) highlighted the use of implementation science for the development of clinical decision support tools for patient care [50]. The LHSs included in this review that best facilitated patient–clinician partnerships ensured that patients were actively and regularly embedded into their system, rather than passive participants who simply received information. For example, Levin et al. [44] developed working groups comprising diverse stakeholders and used surveys and focus groups to continually evaluate the experiences of patients.

#### Incentives

Twenty-one included publications (66%) discussed *Incentives* (Table 2) and four sub-themes were identified within this dimension (Table 3). Fifteen of these papers (71%) covered the sub-theme of *financing*, including financial support for LHS programs, for staff training and capacity building, and financial compensation for patient participation in research and development of LHS processes [2, 31, 37–40, 44, 46, 49, 53, 55, 57, 58, 62, 65]. *Transparency* around decision-making, data usage and management, or the involvement of AI, was discussed in eight papers (38%) [31, 38, 45, 48, 50, 54, 59, 64]. Five papers (24%) discussed the need for *incentives aligned to improve LHS processes* [38, 46, 48, 54, 55], such as using performance indicators that measure the time from evidence release to clinical uptake [38]. Two papers (10%) raised concerns about the *sustainability* of the advances made by the LHS during the COVID-19 pandemic [2, 62], due to limited funding or reimbursement.

#### Continuous learning culture

Twenty-six articles (81%) discussed aspects of a continuous learning culture within their LHS’s response to or planning for a pandemic (Table 2). Four sub-themes were identified within this dimension (Table 3). *Workforce* training, leadership, empowerment, and capacity (*n* = 20, 77%) was seen as crucial to LHS culture, with many papers discussing the importance of having staff with the right skills, training, and education (*n* = 12, 46%) [31, 37, 38, 40, 41, 43–46, 53, 55, 64]. Strong leadership to drive change (*n* = 9, 35%) [2, 31, 38, 40, 47–49, 57, 62], programs to empower staff to implement change (*n* = 5, 19%) [40, 55, 60, 64, 65] and dedicated time and support for staff (*n* = 5, 19%) [38, 46, 48, 49, 62] were discussed as enabling the workforce to operate effectively within an LHS framework. For example, in the USA, the Veterans Affairs Quality Enhancement Research Initiative (QUERI) established the Mentoring Cores and the Advancing Diversity in Implementation Leadership program, which were both designed to enhance the expertise of the workforce in implementing and evaluating quality improvement initiatives within the LHS [40].

Many articles highlighted processes of *continuous refinement and learning*, such as using feedback cycles or observable outcomes to inform care (*n* = 19, 73%) [2, 31, 40, 43–47, 50, 53–55, 57–60, 62, 64, 65]. For example, the Canadian COVID-19 Interdisciplinary Clinical Care Network (PC-ICCN) held bi-weekly meetings with clinical care teams in which data were reviewed and changes to care practices were recommended [44]. The establishment of *communication pathways* (*n* = 13, 50%) [2, 31, 36, 37, 44, 47, 48, 53, 55, 57, 58, 60, 64], such as regular meetings, newsletters, online dashboards, or helplines, and *learning collaborations,* that linked staff with multidisciplinary teams or external collaborations, were also presented as a way of exchanging information and coordinating responses to pandemic events (*n* = 12, 46%) [2, 31, 36, 37, 44, 47, 52, 55, 57–59, 64].

#### Structure and governance

Twenty-three studies (72%) described responses that fell within the *Structure and Governance* dimension of the LHS framework (Table 2). The responses were categorized into five main sub-themes (Table 3). Eighteen studies (78%) referred to *leveraging committees* as part of the LHS’s specific response to the COVID-19 pandemic, either through existing or the formation of new committees [2, 36–38, 40, 44–47, 49, 51, 55, 57, 59–61, 64, 65]. For example, the COVID-19 Evidence Support Team (CEST) in Saskatchewan, Canada, established an oversight committee for their LHS, which included researchers, health policymakers, and emergency operations personnel [2, 59]. Fourteen studies (61%) discussed LHS engagement with *supportive collaborations*, which involved formalizing cooperation with external organizations to support their LHS’s response to the COVID-19 pandemic [2, 40, 44, 45, 49, 52–55, 57, 59, 61, 64, 65]. In 12 studies (52%), LHSs drew on a *national or state-level policy* to support their pandemic response, either by leveraging an existing policy or assisting in the creation of a new government policy [36, 40, 44–47, 50, 54, 55, 61, 64, 65]. Nine studies (39%) discussed either leveraging an existing policy that was created by their organization or creating a new policy that was specifically applicable to their LHS [38–40, 45, 51, 52, 54, 57, 61]. This included the modification of existing ethics approval processes to allow for the rapid uptake of COVID-19 related studies and related decision support systems [57]. Six studies (26%) engaged in *strategic goal setting and planning*, by defining formal targets for their LHS’s pandemic response [37, 38, 40, 47, 55, 57]. Finally, three studies (13%) increased *funding* for research and information technology staff to assist with their response [44, 45, 51].

### The reciprocal relationship between pandemics and LHSs

The included studies revealed a reciprocal relationship between pandemics and LHSs, that is, in some cases, the pandemic accelerated the development of a LHS and in others, the LHS facilitated the response to the pandemic. Twenty-nine (91%) papers discussed a positive interaction between pandemics and LHS development or advancement. The rapidly evolving nature of the pandemic facilitated LHSs advancement of the *Science and Informatics* dimension, increasing real-time capture and synthesis of new evidence into practice through the development and integration of IT systems, such as data warehouses, eHR systems, and dashboards to facilitate decision making (*n* = 19, 66%) [31, 38–42, 46, 47, 50, 51, 54, 55, 57–62, 65]. The pandemic also accelerated the *Continuous Learning Culture* dimension; the principle of rapid change in practice in response to new evidence (“learning while doing” culture) was enhanced and organizational barriers to collaboration and information sharing were reduced through regular meetings with multiple stakeholders (*n* = 19, 66%) [2, 31, 37, 38, 41, 42, 44–47, 52, 55, 57–60, 62–64]. The *Structure and Governance* (*n* = 12, 41%) [37–41, 45, 46, 53, 55, 59, 64, 65] of the LHSs facilitated streamlining of ethics and research processes [38, 41, 45, 64, 65] and the ability to develop and deploy public health strategies [37, 39, 55, 59]. It also enabled working groups that can be commissioned and decommissioned as the course of a pandemic changes [37, 46, 53]. *Incentives* (*n* = 5, 17%) included financial support for the development and continuation of programs [36, 49, 65], alignment of programs with high-value care [54], and transparency of decision-making processes [59]. Two papers (7%) discussed how *Patient–Clinician Partnerships* enhanced the pandemic response, one via the integration of patient feedback in virtual care programs [38] and one through embedding new care practices in usual care to improve equity of access [45].

However, pandemics also presented challenges to the development or advancement of an LHS (*n* = 22, 69%). The pace of the pandemic spread created the need for rapid changes to policies and procedures in limited time frames [37, 38, 46, 57, 58, 61, 64] and brought the added difficulty of managing diverse stakeholders (such as the coordinating local and national level resources). The existing *Structure and Governance* of some LHSs struggled to adjust rapidly to these demands (*n* = 13, 59%) [2, 31, 37, 38, 46, 54, 55, 57, 58, 61, 64]. In addition, strict regulations and legal frameworks either reduced data access for policy making or the ability to implement policy in several systems [46, 52, 54, 65]. Reprioritization of resources also disrupted ongoing research activities, which are a key component of any LHS [37, 61]. Challenges relating to the *Science and Informatics* dimension (*n* = 10, 45%) included some LHSs experiencing problems with IT systems (e.g., eHRs) that were not fit for purpose and struggling to collate new data and evidence, manage data requests, and disseminate information [2, 43, 52, 53, 60] to rapidly use evidence to inform policy and practice changes [47, 53–55, 57, 58].

LHSs’ response to the COVID-19 pandemic at times limited *Patient–Clinician Partnerships* (*n* = 7, 32%). The need to isolate patients and restrict family and carer visits created a particular challenge for LHSs, in which *Patient–Clinician Partnerships* are meant to be central [46, 57]. Lack of communication and difficulties building trust [57, 62] created potential risks for patient harms. The rapid pace of decision-making also meant that patients and families were not involved in co-design of care or programs [38, 49]. Two papers discussed societal issues as challenges to the LHS, including equity of access to care and reacting to political decision-making while attempting to deliver patient-centered outcomes [36, 50].

The goal of creating a *Continuous Learning Culture* (*n* = 5, 23%) was challenged by the volume and speed of changes to the evidence base which caused high workloads and reduced the ability to ensure the latest practices and new health technologies (e.g., new vaccines) were implemented and shared with the community [38, 50, 58, 62]. Disincentives, including lack of transparency around decision making for vaccination campaigns and minimal financial incentives for healthcare workers, were discussed in three papers (14%) [46, 50, 57]. In addition, two publications, which were not classified under the five dimensions, referred to challenges to the sustainability of the progress made, such as the use of committees for rapid research review and financing for newly established programs, during the pandemic [2, 44].

## Discussion

In this rapid review, we synthesized the empirical literature on how LHSs are responding to, and preparing for, health system impacts associated with pandemics and climate change using the five LHS framework dimensions [13, 17]. In line with research on LHSs prior to the COVID-19 pandemic [23, 24, 26, 66, 67], *Science and Informatics* was a central theme in many of the included publications and comprised strategies such as using data management systems (e.g., eHR and clinical dashboards), digital healthcare delivery, and data-driven research projects to rapidly collate and disseminate usable information. The increased focus on data and the rapid generation of knowledge during a public health emergency provided the opportunity for healthcare systems to cultivate a *Continuous Learning Culture* in their pandemic response. Continuous cycles of feedback, improved communication, and strengthening the training and capacity of the healthcare workforce were cited as central to effective LHS functioning during the COVID-19 pandemic. A key benefit of the LHS *Structure and Governance* was the ability to streamline research and ethics approval processes and the ability to rapidly develop and deploy public health strategies [37, 39, 55, 59]. The ability to overcome siloes across healthcare organizations and bring together diverse stakeholders as well as to rapidly commission and decommission working groups was an important component of real-world LHS responses and can be used to respond to future challenges [37, 46, 53].

While the studies included in this review mentioned opportunities for the development of an LHS provided by the pandemic, many reported that they were challenged by outdated or complex *Structure and Governance* and had difficulty integrating new data to rapidly make decisions and implement new findings [37, 38, 46, 52, 54, 57, 61, 64, 65]. The rapidly changing nature of pandemics also appeared to lessen the ability to meet the LHS aspiration of engaged patients, families, and public through *Patient–Clinician Partnerships* due to the necessity to isolate patients and to swiftly implement changes to care and programs [46, 57, 68]. The volume and speed of change created heavy workloads for committees used to review research proposals and evidence emerging from patient outcomes and research. This, in addition to healthcare workforce fatigue, challenged the principle of creating a *Continuous Learning Culture* during the COVID-19 pandemic [38, 50, 53, 58, 62]. These challenges limited the ability of many LHSs to be adaptable, equitable, inclusive, and person focused, all of which are core values of an LHS [68]. This is an important finding as future pandemics are likely, and surges in new types of patients will accompany the predicted increase in climate-related events. Additionally, these impacts will be disproportionately felt in different countries and health systems, depending on their locations and resources. Thus, LHSs need to be “future-proofed” to be able to maintain alignment with their core values in the face of future challenges, and this process needs to account for the diverse needs of health systems. Some of the papers included in this review provide preliminary examples of how this might be achieved by leveraging the rapidly changing nature of a pandemic [38–42]; however, it is likely that alternate strategies will be required for climate related events and future pandemics.

Several LHSs adapted existing LHS frameworks or created new frameworks to meet the unique conditions created by the COVID-19 pandemic [44, 49, 52, 54, 59, 64]. The adapted frameworks refined and operationalized the integration of research into routine healthcare through streamlining ethics approvals and creating standing rapid review committees, as well as drawing on research on systems change, such as implementation science, data science, and quality improvement research [13, 68, 69]. The new frameworks expanded the concept of an LHS to include information and knowledge sharing with the broader health system and the public at local to national levels [52, 54]. This is an important addition to existing frameworks as health systems must operate within the broader societal context, including public health and prevention as well as informing government policy during a pandemic. The fact that nearly 50% of papers did not include a framework suggests the need for a more standardized approach to reporting on and framing LHSs in the literature, which may facilitate the sharing of knowledge across LHSs.

Some papers commented on the need for transparency around decision-making and improved public messaging, as well as the importance of a health literate population. This emphasizes the need for transparency and public trust to facilitate *Patient–Clinician Partnerships*, which continues to be a challenging area for LHSs [17, 23, 70].

There were very few papers in which the focus of the LHS was “long COVID” [31, 36]. This may be because the research on and understanding of long COVID is still emerging [71] and there is typically a lag in publications on new topics.

Perhaps most importantly, our search returned no reports of how an LHS was adapting to or preparing for the health-system effects of climate change. Pandemics and climate change are not mutually exclusive phenomena. Climate change increases the likelihood of new diseases and the emergence of current diseases in new areas (e.g., expansion of vector-borne illnesses) as well as increasing the number and severity of weather-related disasters. As such, climate-related disasters and pandemic waves will cause huge influxes of new and different types of patients into the healthcare system, which must then cope with this increased volume and complexity of care delivery [72]. Thus, while the results of this review describe strategies by which LHSs can be leveraged to respond to pandemics, they have a broader relevance to preparing the health system for the effects of climate change. For example, in many studies, different health organizations worked together to create communities of practice that shared information, rapidly reviewed data and new evidence, and generated guidelines that could be quickly implemented. This strategy is also applicable to managing the immediate effects of climate-related disasters (e.g., floods, bushfires) and outbreaks of new diseases. Additionally, a skilled and appropriately trained and equipped workforce was a key component of many LHS responses to the pandemic, mostly through leveraging leadership structures and facilitating opportunities for staff ongoing training and communication [31, 48, 57]. This is an equally important component of an LHS response to climate change, where severe climate events will disrupt workforce capacity challenging health systems’ ability to adapt to a changing environment. In order to prepare for the increasingly likely consequences of a warming planet, including more frequent natural disasters and pandemics, current and future LHSs will need to evolve to become LHSs 2.0, systems that are not only advancing along an LHS journey (LHS 1.0) but are increasingly equipped to respond to the fast moving and long-term effects brought about by both pandemics and climate change [27]. An LHS 2.0 will not only need to master the skills, capabilities, policies, and engagement required to be an LHS but will need to be even more adroit at change management to be future proofed against the unpredictable shocks associated with climate change [27]. Developing this next level LHS may also facilitate improvement of the environmental sustainability of health systems. Globally, health systems contribute ~ 4–5% of the total greenhouse gas emissions [73, 74]. The principles of LHSs can and should be used to measure, monitor, and mitigate sources of GHG emissions.

While similar LHS responses may in theory apply to both pandemics and climate change, their practical application to climate change may face unique challenges. Many of the studies included in this review reported that the urgent, global, and singular threat posed by COVID-19 facilitated an unprecedented increase in the perceived importance of the LHS and a concomitant unified acceleration in the development of multiple aspects of the LHS in real-world settings. In contrast, climate change is often perceived as a long-term or future threat [75], with impacts that will largely affect “others” [76]. Indeed, it has been suggested that the COVID-19 pandemic suppressed climate change activism and attention in the media and online [77]. Thus, organizations that seek to use the LHS to respond to the health system impacts of climate change will likely first have to overcome this reduced sense of urgency and priority. This issue is also pertinent to the LHS response to COVID-19, as it moves from a pandemic towards an endemic disease and its sense of urgency declines [78–80]. Many studies included in this review describe their immediate responses to COVID-19 or other pandemics, with little mention of sustaining these responses in the absence of a public health emergency or learning from them to prepare for the almost certain onset of future pandemics [44, 59, 80]. Thus, the sustainability of LHS responses to pandemics and climate change is an important area for future consideration.

This review has some limitations. For example, the choice of thematic analysis method (deductive or inductive) has weaknesses and strengths. Deductive analysis, as employed in this review, using a pre-existing framework can facilitate comparison with other studies. However, it may limit new insights that do not conform to the set framework. Inductive approaches tend to have reduced inter-rater reliability and more potential for bias, but may provide more flexibility, allowing for a different understanding of the data. In addition, all reviews may miss publications due to the heterogenous nature of research articles and biases towards publication of specific outcomes. For this review, only studies that were published in English were included, as such, works from some countries may have been inadvertently omitted from the analysis. Publications frequently lag considerably behind real-world progress. Thus, it is likely that the literature included in this review does not describe all LHS responses to the challenges posed by pandemics, particularly the more recent COVID-19 pandemic and treatment of long COVID. Another known issue in the LHS literature is that the use of the term “Learning Health System” or a similar variation is not consistent between countries or health systems [24]. To be included in the review, studies needed to specifically associate their health system or their health system’s responses with an LHS, and for example, we did not include “living guidelines” (a method for rapidly updating clinical practice guidelines [81]) as a search term. Subsequently, health systems emulating LHS principles but that do not explicitly describe themselves as an LHS would not have been captured in this review. The rapid review format used in this review, as proposed by Cochrane, necessitates additional conditions (e.g., the need to define boundaries on the sources of included information and stricter inclusion criteria) to coherently answer the questions of interest [29].

Health system outcomes may also be published in non-peer-reviewed formats, such as reports or webpages, which are more time-consuming to search and therefore would be excluded from rapid reviews. Additionally, outcomes can be published as commentaries or editorials, which commonly describe changes in practice in the absence of comprehensive methods and data. Articles that were excluded from our review for these reasons typically described similar LHS approaches to the studies that were included, for example, many described the formation of working groups or committees [1, 51, 82, 83] and the use of data repositories or warehouses [51, 84–86]. Others described processes of data dashboards and syntheses [1, 51, 83, 86–89], the creation of new guidelines or policy [85, 89], specific staff training [21, 90], and partnerships with other services [21, 82], which were all similar to those described by studies included in our rapid review.

Several recommendations for changes to practice, policy, and research emerged from this review. The included studies provide a sound overview of strategies through which LHSs can respond to the health system impacts of pandemics, and by extension, of climate change. Health systems that seek to use LHS principles to design their response to either of these threats can learn from the collective experience of the studies reported here, which recommend a collective, data-driven approach that is underpinned by clear policy and workforce support. To develop the essential LHS *Continuous Learning Culture,* systems and providers need to learn how to rapidly integrate data and evidence into healthcare delivery. In essence, healthcare systems need to learn how to learn. This requires upskilling of the health workforce in LHS competencies and approaches, embedding evaluations and feedback loops into practice, and breaking down of siloes to foster cross-disciplinary collaboration [19, 25, 40].

The research also reveals that IT systems developed or modified for an LHS, such as new databases [51], registries [56], and open-source software [55, 65], can be used for surveillance of new diseases and threats to human health and health system sustainability [47, 52, 55, 64].

Many of the studies in this review reported opportunities and benefits as well as challenges posed by the COVID-19 pandemic to LHSs. Some studies reported evaluations of the effect of LHS activities on patient outcomes (e.g., reporting lower mortality in patients with COVID-19 in their medical centre after they started using data as an LHS [60], increasing the ability to use observational data to inform patient care [58, 62] and on the speed of uptake of research evidence [60, 63]). It is important for current and future LHS responses to formalize the evaluation of the impact of their responses on both clinical and system outcomes to ascertain the efficacy of this application of LHS principles.

## Conclusions

LHS architecture, in which data is used to rapidly create knowledge and in turn improve practice [18], is well-suited to respond to the uncertainty and rapidly changing conditions of a pandemic and to prepare health systems for the effects of climate change. Despite this potential, there were no included papers that linked LHSs with preparedness for climate change. The LHSs in this review revealed how embracing a continuous learning culture, which integrates new data from patients and research, and employs a skilled and capable workforce, can inform patient care, public policy, and public messaging [59]. The use of IT systems to collect and disseminate information for decision-making also enables LHSs to act as surveillance systems for future pandemics [41, 42] and climate change-related events. There is untapped potential in LHSs to use data to model appropriate system responses to future pandemics and climate change thereby bolstering preparedness planning.

### Supplementary Information

**Additional file1 Tables S1-S4.** **Table S1.** *–* Search strategies. **Table S2.** *–* Data extraction design in REDCap. **Table S3.** *–* Extracted data from included papers. **Table S4.** *–* Quality appraisal assessment.

## Data Availability

Datasets developed and analyzed in this study, including the search strategy, list of the included and excluded studies, extracted data, analysis plans and quality assessment are available in the article or additional files and upon request from the corresponding author. No individual participant data was used for this study.

## Abbreviations

AI: Artificial intelligence
eHR: Electronic health records
eMR: Electronic medical records
JBI: Joanna Briggs Institute Critical Appraisal Checklist for Systematic Reviews and Research Synthesis
LHS: Learning Health System
MMAT: Mixed-Methods Appraisal Tool
SANRA: Scale for the Assessment of Narrative Review Articles
