# Supplementary material for: Progress with the Learning Health System 2.0: a rapid review of Learning Health Systems’ responses to pandemics and climate change
Source: BMC Med. 2024 Mar 22;22:131. doi: 10.1186/s12916-024-03345-8 (PMC10960489; doi:10.1186/s12916-024-03345-8)
Supplement: Supplementary file 1 — Additional file1 Tables S1-S4. Table S1. – Search strategies. Table S2. – Data extraction design in REDCap. Table S3. – Extracted data from included papers. Table S4. – Quality appraisal assessment. [file 12916_2024_3345_MOESM1_ESM.docx]

# Additional File 1:

**Table S1** *–* Search strategies

| **Topic** | **Database: Embase** | **Database: Scopus** | **Database: PubMed** |
| --- | --- | --- | --- |
| Climate change | 1. climate change/ or greenhouse effect/  2. climate change.mp. or climate change/  3. natural disaster.mp. or natural disaster/  4. carbon emission.mp. or carbon emission/  5. climate hazard.mp.  6. ultraviolet radiation.mp. or ultraviolet radiation  7. 1 or 2 or 3 or 4 or 5 or 6  8. learning health system*.mp. or learning health system  9. 7 and 8 | TITLE-ABS-KEY ( "learning health* system*" AND ( "climate change" OR "greenhouse effect" OR "natural disaster" OR "carbon emission" OR "climate hazard*" OR "ultraviolet radiation" OR "climat* variabilit*" ) ) | "learning health* system*" AND ( "climate change" OR "greenhouse effect" OR "natural disaster" OR "carbon emission" OR "climate hazard*" OR "ultraviolet radiation" OR "climat* variabilit*") |
| Pandemics | 1. learning health system*.mp. or learning health system/  2.pandemic/  3. COVID-19.mp. or coronavirus disease 2019/ 4.pandemic*.mp.  5.SARS-COV-2.mp. or Severe acute respiratory syndrome coronavirus 2/  6. 2 or 3 or 4 or 5  7. 1 and 6 | "learning health system*" AND "coronavirus disease 2019" OR covid-19 OR pandemic* OR sars-cov-2 OR "Severe acute respiratory syndrome coronavirus 2" | "learning health system*" AND ("coronavirus disease 2019" OR covid-19 OR pandemic* OR sars-cov-2 OR "Severe acute respiratory syndrome coronavirus 2") |

Table S2 *–* Data extraction design in REDCap

| **Field Name** | **Field Type** | **Field Label** | **Choices** |
| --- | --- | --- | --- |
| **Basic information** | | | |
| record_id | text | Record ID |  |
| reviewer_id | dropdown | Reviewer |  |
| id | dropdown | First author (year) |  |
| author_country | text | First author's country |  |
| study_design | checkbox | Study design | 0, Empirical \| 1, Case study \| 2, Narrative descriptive \| 3, Theoretical \| 4, Other |
| other_design | text | Other study design |  |
| study_aim | notes | Study aim: |  |
| **Health setting information** | | | |
| health_sector | radio | Health sector | 0, International \| 1, National \| 2, State \| 3, Community \| 4, Other \| 5, N/A |
| other_sector | text | Other health sector |  |
| sector_na_reason | text | Reason for N/A |  |
| health_setting | checkbox | Health setting | 0, Hospital network \| 1, Hospital \| 2, Primary care \| 3, Aged care \| 4, Community service \| 5, Other \| 6, Not reported \| 7, N/A |
| other_setting | text | Other health setting |  |
| health_setting_na | text | Reason for setting N/A |  |
| setting_location | checkbox | Health setting location | 0, Urban \| 1, Regional \| 2, Rural \| 3, Remote \| 4, N/A |
| oecd | checkbox | Health setting OECD classification | 0, Low income \| 1, Low-middle income \| 2, Middle-upper income \| 3, High income |
| setting_country | text | Health system country |  |
| **Pandemic information** | | | |
| pandemic_type | checkbox | Pandemic type | 0, Current / COVID-19 \| 1, Future pandemic \| 2, Other pandemic |
| other_pandemic | text | Other pandemic type |  |
| **Summary of paper** | | | |
| paper_summary | notes | Summary of key points (2 - 3 sentences) |  |
| **LHS Information** | | | |
| lhs_stage | checkbox | LHS Stage (in paper) | 0, Implemented (past / present) \| 1, Future |
| lhs_name | text | LHS Name |  |
| lhs_focus_1 | text | LHS Focus (e.g., Epilepsy LHS, Wounds LHS) |  |
| lhs_definition | notes | LHS Definition |  |
| lhs_def_refs | notes | LHS Definition References |  |
| lhs_framework | notes | LHS Framework |  |
| **LHS Dimensions** | | | |
| lhs_dimensions | checkbox | LHS Dimensions | 0, Science and informatics \| 1, Patient clinician partnerships \| 2, Incentives \| 3, Continuous learning culture \| 4, Structure and governance |
| science | notes | Science and informatics: Real-time access to knowledge, digital capture of care experience |  |
| partnerships | notes | Patient clinician partnerships: Engaged-empowered patients |  |
| incentives | notes | Incentives: Incentives aligned for value, Full transparency |  |
| learning_culture | notes | Continuous learning culture: Leadership-instilled culture of learning, Supportive system competencies |  |
| governance | notes | Structure and governance: Policies, governance, and regulations aligned to facilitate research, collaboration, and learning |  |
| **Benefits / challenges** | | | |
| benefits | notes | Benefits of pandemic / climate change to LHS |  |
| challenges | notes | Challenges of pandemic to LHS |  |
| **Other information** | | | |
| other_notes | notes | Other notes |  |
| snowball_refs | notes | Potential papers to snowball references  Copy reference |  |
| snowball_reasons | notes | Potential papers to snowball reasons |  |

**Table S3** *–* Extracted data from included papers

| **First author (year)** | **Study aim** | **Summary of key points** | **LHS Name** | **Pandemic (Current or future)** |
| --- | --- | --- | --- | --- |
| Allen 2021 | To develop a roadmap for organizations that want to establish an LHS program, understand how LHS core components relate to one another when operationalized in practice, and evaluate and improve their progress. | "KPWA LHS Logic Model provides a broad set of constructs relevant to LHS programs, depicts their relationship to LHS operations, harmonizes terms across models, and offers measurable operationalizations of each construct to guide other health systems. The model identifies essential LHS inputs, provides transparency into LHS activities, and defines key outcomes to evaluate LHS processes and impact. We provide reflections on the most helpful components of the model and identify areas that need further improvement using illustrative examples from deployment of the LHS model during the COVID-19 pandemic." | Kaiser Permanente Washington | Current (COVID-19) |
| Anderson 2022 | "This experience report details three characteristics of institutional-level learning system development, derived from the experience of the UAB (University of Alabama at Birmingham) COVID-19 CORE including: (a) identifying network contributors and components, (b) building the institutional network, and (c) diversifying network capabilities." | "This experience report details three principal characteristics of the UAB COVID-19 CORE LHS development: (a) identifying network contributors and components; (b) building the institutional network; and (c) diversifying network capabilities."  The utilization of adapted LHS framework as a guiding principle to analyze and critically evaluate LHS form and functions is crucial to achieve an effective integrated health service outcomes and research response. | The UAB COVID-19 Collaborative Outcomes Research Enterprise (CORE) | Current (COVID-19) |
| Atkins 2022 | "In this review we reflect on more than 10 years of experience at the Veterans Health Administration, the largest integrated healthcare system in the United States, in developing, testing, and implementing such models at scale. We report lessons from the implementation of national risk prediction models and suggest an agenda for research." | The article reviews the LHS at the Veterans Health Administration over the past 10 years. It details specific impacts related to the LHS functioning during COVID-19, and puts forward an agenda for future research. | US Department of Veterans Affairs (VA); Veterans Health Administration | Current (COVID-19) |
| Bakshi 2021 | "In this article, we describe how mature population health programs in a learning health system have been rapidly leveraged to address the challenges of the pandemic." | Partners Healthcare System (PHS) leveraged their LHS structure to design and implement public health responses to the COVID-19 pandemic. These responses were focused on equitable care. | Partners Healthcare System (PHS) | Current (COVID-19) |
| Braganza 2022 | "To describe the design and impact of a systematic, enterprise-wide process for engaging US Department of Veterans Affairs (VA) leadership in prioritizing scarce implementation and evaluation resources." | The VA Quality Enhancement Research Initiative (QUERI) started in 2017, and used its principles and strategies to respond to the COVID-19 pandemic. The initiatives remit was to prioritize and fund initiatives to increase evidence-based practice in the VA. | Veterans Affairs Quality Enhancement Research Initiative (QUERI) | Current (COVID-19) |
| Brunet 2022 | "Our objective is to analyze the value of innovative solutions implemented by a learning health system, including an academic health center, to reconnect the Quebec health system to population needs, integrating the five elements to improve population health." | The Centre Hospitalier del'Universite de Montreal (CHUM) is an academic health center that is embedded into the University of Montreal's university network, that brings together 14 health institutions. These institutions aim to use a LHS approach to improve care, and recently leveraged this approach to respond to the COVID-19 pandemic. | University of Montreal's university network | Current (COVID-19) |
| Cassidy 2022 | "To use a LHS framework to identify assets and gaps in health system pandemic planning and response during the initial stages of the COVID-19 pandemic at a single Canadian Health Centre" | This paper uses administrative, textual, and qualitative data to examine "rapid learning and change during the COVID-19 pandemic" within the health center. The paper uses an LHS framework to map this data and identify any gaps and strategies to help inform health system change. | Health Centre (For the purpose of this study, the Health Centre is the defined health system.) | Current (COVID-19) |
| Daniel 2022 | "In this context, the objective of this paper is to describe the AP-HP Health Data Space (AHDS) and the IT services that enable the reuse of observational and interventional health data to support clinical and translational research and to move towards a learning health system" | The ADHS was a pre-existing LHS that prior to the COVID-19 pandemic had put various policies and strategies into place to move towards becoming an LHS. The COVID-19 pandemic enabled the ADHS to leverage its LHS competencies to appropriately respond to the pandemic. | Assistance Publique - HÃ´pitaux de Paris - Health Data Space | Current (COVID-19) |
| Dash 2022 | "This article provides a background on the current state of observational data generation in institutional guideline creation and details our institution's experience in creating a novel workflow to (1) demonstrate the value of such a workflow, (2) demonstrate a real-world example, and (3) discuss difficulties encountered and future directions." | Development of "near real-time clinical decision support at a population level" for the treatment of COVID-19 positive patients in hospital using EHR and observational data. Selected four questions designed by "multidisciplinary team consisting of practicing clinicians, electronic medical record reporting specialists, data scientists, and clinical informaticians (CIs)." | Stanford Hospital | Current (COVID-19) |
| English 2021 | "To illustrate how our pediatric and neonatal research has benefited from efforts to develop an early form of LHS in Kenya." | "…To illustrate how our pediatric and neonatal research has benefited from efforts to develop an early form of LHS in Kenya." | Clinical Information Network (CIN) | Current (COVID-19) |
| Foraker 2021 | "Herein, we describe lessons learned and a framework to address these needs, which focus on: (a) identifying and filling technology "gaps"; (b) pursuing collaborative design of data sharing requirements and transmission mechanisms; (c) facilitating cross-domain discussions involving legal and research compliance; and (d) establishing or participating in multi-institutional convening or coordinating activities." | Washington University and BJC HealthCare developed a LHS informed strategy to address the challenges of the COVID-19 pandemic. They overcame multiple barriers and described these in the article. They attempted to expand their model to other healthcare services in the area but were unsuccessful. They describe recommendations for an optimal COVID-19 LHS response. | Washington University and BJC HealthCare | Current (COVID-19) |
| Fox 2021 | Research questions are as follows:   - "Can we create executable models of best practice in the care of COVID-19 patients? - Can this demonstrate and maintain a standardised model of good practice ("reference model")? - How useful is the OpenClinical knowledge sharing framework for empowering clinicians to critique and improve models of decision making and care across the patient journey? - Is it possible to adapt components of the model for use in different clinical settings or in local variants of care pathways? - What is the potential for combining knowledge engineering methods with techniques from data science (e.g., statistical analysis, data mining and machine learning)? | This paper presents a case study of OpenClinical, which is a platform for the knowledge-to-data cycle within an LHS. The paper also focuses on PROforma, which is a machine learning model which can be used within the rapid learning system to model clinical guidelines. | OpenClinical | Current (COVID-19) |
| Groot 2022 (2) | "The primary aim of CEST was to produce and sustain the best available COVID-19 evidence to facilitate decision-making in Saskatchewan, Canada. To achieve this objective, four provincial organizations partnered to establish a single, data-driven system… In this report, we will describe how the four provincial institutions collaborated to (a) respond to the need for a single reliable COVID-19 evidence database, (b) maintain a continuously updated source of COVID-19 evidence, (c) facilitate COVID-19 decision-making by providing this evidence to administrators, clinicians, and policymakers, and (d) initiate a Learning Health System in response to a difficult and fast-moving crisis." | Key focus of the LHS in this paper:   - Developed a working group to review COVID-19 evidence. - No consumer involvement due to time and budget constraints. - Prioritised questions for rapid review. - Created a dashboard with the evidence reviews. - Problem with the quality of the early evidence. - Raised questions about the financial sustainability of the LHS. | COVID-19 Evidence Support Team (CEST) | Current (COVID-19) |
| Groot 2022 (59) | "The purpose of this article is to describe a formative evaluation of the CEST initiative to assess its implementation and impact, and to explore how it might develop into a sustainable LHS for Saskatchewan." | In 2020, the University of Saskatchewan (USASK), Saskatchewan Health Authority (SHA), Health Quality Council (HQC), and Ministry of Health (MoH) combined to create CEST - the COVID-19 Evidence Support Team. This article reports a formative evaluation of the initiative by interviewing 13 key informants, who were responsible for decision-making during the pandemic. | COVID-19 Evidence Support Team (CEST) | Current (COVID-19) |
| Gustavson 2022 | "This article provides an overview of our emerging LHS approach to the study of long COVID care that is fostering innovation and adaptability within the VHA." | The VHA formed a multidisciplinary workgroup to develop communication and collaborations amongst healthcare institutions, to identify gaps in care and research to create a foundation for cocreating knowledge about the safe and effective management of long COVID sequelae. The following innovations influenced by community of practice group: changes in care delivery, engage in active outreach with veterans and provide infrastructure to sustain long COVID clinics. | Veterans Health Administration (VHA) | Current (COVID-19), Long COVID |
| Hunt 2021 | "This paper describes the realization of one COVID-19-specific digital health solution: COVID-19 Clinical Rounds." | An established telehealth initiative created a new virtual education resource, the Project ECHO COVID-19 Clinical Rounds Initiative. This initiative created platforms to establish an international peer-learning network to share clinical experiences in dealing with the COVID-19 pandemic. | Project ECHO COVID-19 Clinical Rounds Initiative | Current (COVID-19) |
| Levin 2022 | "Herein, we describe the COVID-19 Interdisciplinary Clinical Care Network (PC-ICCN) and data infrastructure developed to support ongoing clinical care and research." | "The PC-ICCN emerged through collaboration among over 60 clinical specialists, researchers, patients, and health administrators. [It] was conceived in May 2020 in recognition of an emerging need for specialized coordinated care for a subset of post COVID-19 patients with persistent symptoms (now recognized as long-haul COVID, long COVID, or Post Acute Sequelae of COVID-19, PASC). Modelled as an LHS, the PC-ICCN evolved iteratively to address patient care goals, facilitate clinical collaboration, and enhance research integration." | COVID-19 Interdisciplinary Clinical Care Network (PC-ICCN) | Current (COVID-19) |
| McCreary 2022 (60) | "To describe how the University of Pittsburgh Medical Center (UPMC) committed to a rapid learning health system (LHS) model to respond to the COVID-19 pandemic… We evaluated the influence of the UPMC COVID-19 therapeutics Committee on change in COVID-19 clinical practice by time series plotting of the prevalence of in-hospital use of selected medications in relation to internal analyses and key scientific publications and regulatory approvals routinely reviewed by the committee.” | In response to COVID-19 University of Pittsburgh Medical Center established the multidisciplinary COVID-19 Therapeutics Committee. This paper discusses how the committee evaluated RCTs, publications, and FDA drug approvals and then implemented these into practice for treating COVID-19 patients. They embedded the guidelines in the EMR system. | University of Pittsburgh Medical Center (UPMC) | Current (COVID-19) |
| McCreary 2022 (61) | "We set two objectives: to equitably treat as broad a proportion of mAb-eligible patients as possible, and to compare the effectiveness between mAbs overall and over time as SARS-CoV-2 variants emerged.”  “We report the first results of the OPTIMISE-C19 (OPti-mizing Treatment and Impact of Monoclonal antIbodieS through Evaluation for COVID-19) trial, evaluating monoclonal antibody use through June 25, 2021" | This paper "reports the first results of the OPTIMISE C-19 (Optimizing Treatment and Impact of the Monoclonal antIbodieS through Evaluation for COVID-19) trial, evaluating monoclonal antibody use through June 25, 2021". This mAb treatment trial was developed within an LHS framework, such that "trial procedures were embedded within routine care". | OPTIMISE-C19 | Current (COVID-19) |
| McCreary 2022 (45) | "We sought to rapidly launch an adaptive platform trial with the goals of enhancing access to treatment, regardless of geography and socioeconomic status, and evaluating comparative efficacy and safety of available mAbs... We describe trial infrastructure, lessons learned, and future directions for a culture of learning while doing."" | This is an empirical paper that details the OPTIMISE-C19 trial infrastructure, priorities under an LHS framework, and plans for implementation. Some learnings from the trial are also presented. | OPTIMISE-C19 (OPtimizing Treatment and Impact of Monoclonal antIbodieS through Evaluation for COVID-19) | Current (COVID-19) |
| Millimouno 2023 | "The main objective of this study was to analyze how Guinea's health system has learnt from the response to outbreaks between 2014 and 2021…Specifically, we sought to (1) identify lessons learnt and whether and how the health system incorporated these lessons into its learning process and the response to future outbreaks, drawing on the literature, (2) describe learning that took place at different health system levels from the stakeholders' perspective, (3) determine enablers and barriers to LHS from stakeholders' perspective and (4) formulate recommendations for improvement of learning within the health system from the perspective of stakeholders." | This paper focuses on lessons learnt from a number of outbreaks in Guinea such as Ebola (EVD) outbreak in 2014-2016, Measles, Lassa Fever, COVID-19, 2021 EVD and Marburg virus disease. "The health system performed well and achieved encouraging and better outbreak response outcomes over time with learning that occurred." | District Team (piloted in Guinea and Benin 2016-2017); Guinea health system | Current (COVID-19), Future |
| Polancich 2021 | "The purpose of this article is to discuss the authors' findings from a descriptive analysis of Hospital Acquired Pressure Injury (HAPI) development among patients who were COVID-19 positive, comparing HAPI characteristics at two time points to reflect the organizational learnings that transpired as the pandemic progressed." | The use of LHS framework to understand the impact of COVID-19 pandemic on the incidence of HAPIs. At the beginning of the pandemic, the Wound, Ostomy and Continence Team quickly utilized teletechnology in evaluating and monitoring patients. The role of nursing leadership team was critical to provide timely responses to improvement interventions. | N/A | Current (COVID-19) |
| Ros 2021 | To describe "a proposed comprehensive standards-based systems approach and data-driven framework for collection, management, and analysis of high-quality data [that] will inform decisions in managing clinical responses and social measures to overcome the Covid-19 global pandemic and to prepare for future public health crises" | This article presents recommendations from three countries: Italy, Spain, and the USA, about the optimal LHS response to COVID-19. The approach is underpinned by systems theory, and their model demonstrates how data can be integrated into workflows to identify and respond to cases of COVID-19. The model emphasizes the importance of adequate data, data standards and policy, and of new technologies such as contact tracing apps. | A systems approach to data management for Covid-19 | Current (COVID-19) |
| Saleh 2021 | "The paper informs on some of the steps taken by the Centre regarding learning from the Lassa fever outbreak and the COVID-19 pandemic in Nigeria." | The importance of documentation and knowledge management system was a key point from this paper. "Being an annual event, the NCDC recognized that response to Lassa fever outbreaks needed to be adapted to put the country in a ‘prepared’ mode, as against the ‘reactive’ mode." | The Nigeria Centre for Disease Control; the Surveillance Outbreak Response Management and Analysis System (SORMAS) | Current (COVID-19), Future |
| Sheikh 2021 | "The COVID-19 pandemic has offered an opportunity to assess how well equipped the UK is to leverage health information technology and apply the principles of a national learning health and care system in response to a major public health shock." “We begin by discussing how the UK has leveraged health information technology (HIT) in response to the COVID-19 pandemic and then focus on the major priorities and opportunities to strengthen HIT across UK." | Theoretical paper discussing how technology can be used to further the development of NHS into an LHS. This paper has some examples of learning but mostly theoretical discussion of different needs (technology, AI, regulatory, patient trust). | NHS | Current (COVID-19) |
| Sivan 2022 | "To optimize all aspects of long COVID care in the UK, including access to services, care pathways and practices and equity." | LOCOMOTION aims to optimize all aspects of long COVID care in the UK, including quality improvement collaborative, experience-based co-design, outcome measurement and learning health systems. This study involves patients, frontline clinicians, specialist long COVID clinics and integrated long COVID services across primary, secondary and community care. | LOng COvid Multidisciplinary consortium Optimising Treatments and servIces acrOss the NHS (LOCOMOTION) | Current (COVID-19), Long COVID |
| Tai-Seele 2022 | "This brief study reports a rapid-cycle learning project testing the wording of messages, and the digital means with which they were sent, email vs Short Message Service (SMS), aimed at encouraging adoption [of digital exposure notification approaches] among UC San Diego Health patients." | This is an empirical paper that tests different communication approaches of an exposure notification phone tool. This is employed using principles of a rapid learning cycle, which maps onto an LHS. | UC San Diego Health System | Current (COVID-19) |
| UPMC REMAP-COVID Group 2021 | "The Randomized Embedded Multifactorial Adaptive Platform for COVID-19 (REMAP-COVID) trial is a global adaptive platform trial of hospitalized patients with COVID-19. We describe implementation at the first US site, the UPMC health system, and offer recommendations for implementation at other sites." | "REMAP-CAP is a global adaptive platform trial of patients with severe community-acquired pneumonia (CAP) admitted to the intensive care unit (ICU) that was launched in 2016". The platform was adapted to managed COVID-19 patient trials. | University of Pittsburgh Medical Centre Learning While Doing Program | Current (COVID-19) |
| Vahidy 2021 | "We share our experience and provide a framework for assembling and organizing multidisciplinary resources, structuring and regulating research needs, and developing a single source of truth (SSoT) for COVID-19 research by applying fundamental principles of health care digitization, in the context of LHC systems across a complex health care organization." | In response to COVID-19 Houston Methodist (HM) created a retrospective research task force (RRTF) "to prioritize and streamlineCOVID-19 observational research." They created a COVID-19 Surveillance and Outcomes Registry (CURATOR), "a relational structured query language database that is directly populated with data from electronic health records via largely automated extract, transform, and load procedures". CURATOR has IRB approval. | Retrospective research task force (RRTF) | Current (COVID-19) |
| van Rensburg 2022 | "The aim of this paper is to share our experiences in applying learning health systems (LHS) thinking to the co-development of an intervention improving an integrated response to COVID-19 and tuberculosis in a South African district." | The study illustrates adoptions of coproduced intervention by a community learning in the area of TB prevention and treatment within the context of the COVID-19 pandemic using LHS approach in the district of Amajuba in South Africa. "The use of theory of change further helps to strategically steer health systems strengthening for integrated, person-centered TB, mental health and COVID-19 care, within the broader frame of a cyclical LHS approach." Adapting the program to the pandemic and integrating COVID-19 with TB care was also a key point of this article. | N/A | Current (COVID-19), Tuberculosis |
| Vinson 2021 | "How did a Learning Network leadership team use the network's existing infrastructure to support the network community during the COVID-19 response? What can this case teach us about the infrastructural aspects of Learning Health Systems?" | "The Epilepsy Learning Healthcare System (ELHS) was founded in 2018 as part of a pilot initiative to build Learning Health Systems that was supported by the Patient-Centered Outcomes Research Institute (PCORI) and led by the Anderson Center for Health Systems Excellence at Cincinnati Children's Hospital Medical Centre." At the beginning of 2020, the ELHS pivoted to address the challenges posed by the pandemic. This article describes how the ELHS used existing structures to implement their pandemic response. | Epilepsy Learning Healthcare System | Current (COVID-19) |
| Wood 2021 | "This report describes the ASH Research Collaborative and its primary components, the Data Hub and the Sickle Cell Disease Clinical Trials Network (SCD CTN). The development and implementation of the initiative's methods for data collection and tools for users of the data are reviewed, and initial use cases for the generation of real-world evidence (RWE) and the improvement of clinical care are discussed. The report concludes with directions for the future evolution of the ASH Research Collaborative." | "The ASH Research Collaborative was founded as a nonprofit organization by the American Society of Hematology in 2018, to improve the lives of people affected by blood diseases by enhancing research and clinical practice. The Data Hub aggregates curated data from multiple sites for a variety of uses by researchers, providers, and other stakeholders, while the SCD CTN engages institutions to introduce efficiencies in multicenter clinical research." This article details the Collaborative, and briefly describes the way that it pivoted to respond to COVID-19. | ASH Research Collaborative | Current (COVID-19) |

Table S4 *–* Quality appraisal assessment

| **Reference** | **Quality Assessment** | **Tool used**  **(MMAT, SANRA, JBI)*** |
| --- | --- | --- |
| Allen C, Coleman K, Mettert K, Lewis C, Westbrook E, Lozano P. A roadmap to operationalize and evaluate impact in a learning health system. Learn Health Syst. 2021;5(4):e10258. | High | SANRA |
| Anderson JL, Reamey RA, Levitan EB, I MA, M SA, Fletcher FE, et al. The University of Alabama at Birmingham COVID-19 Collaborative Outcomes Research Enterprise: Developing an institutional learning health system in response to the global pandemic. Learn Health Syst. 2022;6(2):e10292. | Moderate | MMAT |
| Atkins D, Makridis CA, Alterovitz G, Ramoni R, Clancy C. Developing and implementing predictive models in a Learning Healthcare System: Traditional and artificial intelligence approaches in the Veterans Health Administration. Annu Rev Biomed Data Sci. 2022;5:393-413. | High | SANRA |
| Bakshi S, Schiavoni KH, Carlson LC, Chang TE, Flaster AO, Forester BP, et al. The essential role of population health during and beyond COVID-19. Am J Manag Care. 2021;27(3):123-8. | Low | MMAT |
| Braganza MZ, Pearson E, Avila CJ, Zlowe D, Øvretveit J, Kilbourne AM. Aligning quality improvement efforts and policy goals in a national integrated health system. Health Serv Res. 2022;57(S1):9-19. | High | SANRA |
| Brunet F, Malas K, Pomey M-P. Reconnecting health through innovation. Healthc Manage Forum. 2022;35(6):344-8. | Moderate | SANRA |
| Cassidy C, Sim M, Somerville M, Crowther D, Sinclair D, Elliott Rose A, et al. Using a learning health system framework to examine COVID-19 pandemic planning and response at a Canadian Health Centre. PLoS One. 2022;17(9):e0273149. | High | MMAT |
| Daniel C, Paris N, Pierre O, Griffon N, Breant S, Orlova N, et al. AP-HP Health Data Space (AHDS) to the Test of the Covid-19 Pandemic. Stud Health Technol Inform. 2022;294:28-32. | Moderate | SANRA |
| Dash D, Gokhale A, Patel BS, Callahan A, Posada J, Krishnan G, et al. Building a learning health system: Creating an analytical workflow for evidence generation to inform institutional clinical care guidelines. Appl Clin Inform. 2022;13(1):315-21. | Moderate | MMAT |
| English M, Irimu G, Akech S, Aluvaala J, Ogero M, Isaaka L, et al. Employing learning health system principles to advance research on severe neonatal and paediatric illness in Kenya. BMJ Glob Health. 2021;6(3). | Moderate | MMAT |
| Foraker RE, Lai AM, Kannampallil TG, Woeltje KF, Trolard AM, Payne PRO. Transmission dynamics: Data sharing in the COVID-19 era. Learn Health Syst. 2021;5(1):e10235. | Moderate | SANRA |
| Fox J, Khan O, Curtis H, Wright A, Pal C, Cockburn N, et al. Rapid translation of clinical guidelines into executable knowledge: A case study of COVID-19 and online demonstration. Learn Health Syst. 2021;5(1):e10236. | Moderate | MMAT |
| Groot G, Baer S, Badea A, Dalidowicz M, Yasinian M, Ali A, et al. Developing a rapid evidence response to COVID-19: The collaborative approach of Saskatchewan, Canada. Learn Health Syst. 2022;6(1):e10280. | High | MMAT |
| Groot G, Witham S, Badea A, Baer S, Dalidowicz M, Reeder B, et al. Evaluating a learning health system initiative: Lessons learned during COVID-19 in Saskatchewan, Canada. Learn Health Syst. 2022;7(3):e10350. | High | MMAT |
| Gustavson AM, Purnell A, Adly M, Awan O, Bräu N, Braus NA, et al. A Learning Health System Approach to Long COVID Care. Federal practitioner : for the health care professionals of the VA, DoD, and PHS. 2022;39(7):310-4. | High | MMAT |
| Hunt RC, Struminger BB, Redd JT, Herrmann J, Jolly BT, Arora S, et al. Virtual peer-to-peer learning to enhance and accelerate the health system response to COVID-19: The HHS ASPR Project ECHO COVID-19 Clinical Rounds Initiative. Ann Emerg Med. 2021;78(2):223-8. | Moderate | SANRA |
| Levin A, Malbeuf M, Hoens AM, Carlsten C, Ryerson CJ, Cau A, et al. Creating a provincial post COVID-19 interdisciplinary clinical care network as a learning health system during the pandemic: Integrating clinical care and research. Learn Health Syst. 2022:e10316. | Moderate | SANRA |
| McCreary EK, Kip KE, Bariola JR, Schmidhofer M, Minnier T, Mayak K, et al. A learning health system approach to the COVID-19 pandemic: System-wide changes in clinical practice and 30-day mortality among hospitalized patients. Learn Health Syst. 2022;6(3):e10304. | High | MMAT |
| McCreary EK, Bariola JR, Minnier T, Wadas RJ, Shovel JA, Albin D, et al. Launching a comparative effectiveness adaptive platform trial of monoclonal antibodies for COVID-19 in 21 days. Contemporary Clinical Trials. 2022;113:106652. | Moderate | SANRA |
| McCreary EK, Bariola JR, Minnier TE, Wadas RJ, Shovel JA, Albin D, et al. The comparative effectiveness of COVID-19 monoclonal antibodies: A learning health system randomized clinical trial. Contemp Clin Trials. 2022;119:106822. | High | MMAT |
| Millimouno TM, Meessen B, Put WVD, Garcia M, Camara BS, Christou A, et al. How has Guinea learnt from the response to outbreaks? A learning health system analysis. BMJ Glob Health. 2023;8(2). | High | MMAT |
| Polancich S, Hall AG, Miltner R, Poe T, Enogela EM, Montgomery AP, et al. Learning during crisis: The impact of COVID-19 on hospital-scquired pressure injury incidence. J Healthc Qual. 2021;43(3):137-44. | High | MMAT |
| Ros F, Kush R, Friedman C, Gil Zorzo E, Rivero Corte P, Rubin JC, et al. Addressing the Covid-19 pandemic and future public health challenges through global collaboration and a data-driven systems approach. Learn Health Syst. 2021;5(1):e10253. | Moderate | SANRA |
| Saleh F, Popoola BO, Arinze C, Elisha AA, Dan-Nwafor C, Olajide L, et al. Adapting public health response through lessons learnt: Nigeria’s experience from Lassa fever and COVID-19. BMJ Glob Health. 2022;7(Suppl 7). | Moderate | MMAT |
| Sheikh A, Anderson M, Albala S, Casadei B, Franklin BD, Richards M, et al. Health information technology and digital innovation for national learning health and care systems. The Lancet Digital health. 2021;3(6):e383-e96. | High | SANRA |
| Sivan M, Greenhalgh T, Darbyshire JL, Mir G, O'Connor RJ, Dawes H, et al. LOng COvid Multidisciplinary consortium Optimising Treatments and servIces acrOss the NHS (LOCOMOTION): Protocol for a mixed-methods study in the UK. BMJ Open. 2022;12(5):e063505. | Moderate | MMAT |
| Tai-Seale M, May N, Sitapati A, Longhurst CA. A learning health system approach to COVID-19 exposure notification system rollout. Learn Health Syst. 2022;6(2):e10290. | High | MMAT |
| UPMC Remap-Covid Group. Implementation of the Randomized Embedded Multifactorial Adaptive Platform for COVID-19 (REMAP-COVID) trial in a US health system-lessons learned and recommendations. Trials. 2021;22(1):100. | High | SANRA |
| Vahidy F, Jones SL, Tano ME, Nicolas JC, Khan OA, Meeks JR, et al. Rapid response to drive COVID-19 research in a Learning Health Care System: Rationale and design of the Houston Methodist COVID-19 Surveillance and Outcomes Registry (CURATOR). JMIR Med Inform. 2021;9(2):e26773. | Moderate | SANRA |
| van Rensburg AJ, Petersen I, Awotiwon A, Bachmann MO, Curran R, Murdoch J, et al. Applying learning health systems thinking in codeveloping integrated tuberculosis interventions in the contexts of COVID-19. BMJ Glob Health. 2022;7(10). | Moderate | MMAT |
| Vinson AH. Putting the network to work: Learning networks in rapid response situations. Learn Health Syst. 2021;5(1):e10251. | Moderate | SANRA |
| Wood WA, Marks P, Plovnick RM, Hewitt K, Neuberg DS, Walters S, et al. ASH Research Collaborative: A real-world data infrastructure to support real-world evidence development and learning healthcare systems in hematology. Blood Adv. 2021;5(23):5429-38. | High | SANRA |

* MMAT = Mixed-Methods Appraisal Tool (33), SANRA = Scale for the Assessment of Narrative Review Articles (SANRA) (34), JBI =Joanna Briggs Institute critical appraisal checklist for systematic reviews and research synthesis (35).
